# A Paradigm Shift in the Management of Atherosclerosis: Protective Role of Sirtuins in Atherosclerosis

**DOI:** 10.7759/cureus.12735

**Published:** 2021-01-16

**Authors:** Ijeoma A Toulassi, Usama A Al Saedi, Sai Dheeraj Gutlapalli, Sujan Poudel, Varshitha Kondapaneni, Mehwish Zeb, Ivan Cancarevic

**Affiliations:** 1 Pathology, California Institute of Behavioral Neurosciences & Psychology, Fairfield, USA; 2 Dentistry, California Institute of Behavioral Neurosciences & Psychology, Fairfield, USA; 3 Internal Medicine, California Institute of Behavioral Neurosciences & Psychology, Fairfield, USA; 4 Family Medicine, California Institute of Behavioral Neurosciences & Psychology, Fairfiled, USA; 5 Psychiatry and Behavioral Sciences, California Institute of Behavioral Neurosciences & Psychology, Fairfiled, USA; 6 Internal Medicine, California Institute of Behavioral Neurosciences & Psychology, Fairfiled, USA; 7 Internal Medicine/Pediatrics, California Institute of Behavioral Neurosciences & Psychology, Fairfield, USA; 8 Pediatrics, Khyber Teaching Hospital, Peshawar, PAK

**Keywords:** atherosclerosis, sirtuins, cardiovascular disease, antiinflammatory, ldl cholesterol, cellular senescence, ros

## Abstract

Facing the rise of an aging population and age-related pathologies such as atherosclerosis will continue to be some of the biggest challenges encountering health care. Regardless of considerable advancements in management and prevention to deal with atherosclerosis and other related pathologies. The current guidelines for preventing and managing atherosclerotic diseases are lifestyle changes, blood pressure control, blood glucose control, and lipid control. There has been an increase in pre-clinical studies regarding the effects of sirtuins on atherosclerosis and this review aims to highlight the benefits of sirtuins in atherosclerosis. We did an extensive search using the PubMed database with the medical subject headings (MeSH) keywords “sirtuin'' and “atherosclerosis.” The reviewed literature reported that sirtuins prevent and ameliorate atherosclerosis by halting inflammation, apoptosis, oxidative stress, and regulating low-density lipoprotein (LDL) cholesterol. Sirtuin 1 (SIRT1) and sirtuin 6 (SIRT6) inhibit the RELA component of NF-kB, thus suppressing inflammation, SIRT1 inhibits p53 by deacetylation, and the latter stabilize telomeres thus preventing apoptosis and cell death. Sirtuin 3 (SIRT3) inhibits oxidative stress by driving the production of reduced glutathione. Sirtuin 2 (SIRT2) regulates LDL cholesterol by inhibiting pcsk9, increasing LDL receptors on the cell surface of hepatocytes. A combination of these effects of sirtuins in the endothelial cells suggests sirtuins are anti-atherogenic and could revolutionize the standards for the management of atherosclerosis. This article also emphasizes the need for future research on human cells or subjects rather than animal subjects.

## Introduction and background

Atherosclerosis comes from the Greek words “athero” meaning “gruel” and “sclerosis” meaning “hardening” [1]. Atherosclerosis is the underlying cause of coronary, cerebral, and peripheral vascular diseases. It constitutes significantly more morbidity and mortality in the western world than any other disorder and an increasing number of deaths in developing countries [1-3]. According to the Framingham Heart study, risk factors for atherosclerosis development include age, sex, total cholesterol, high-density lipoprotein, smoking status, and systolic blood pressure [4]. Atherosclerosis is a multistage process consisting of chronic inflammation, endothelial injury, modified low-density lipoprotein (LDL), T-cell, and monocyte-derived macrophages. The macrophages proliferate and ingest modified LDL forming foam cells, which promote plaque formation and subsequent plaque rupture [1]. Cellular senescence due to chronological aging, reactive oxidative species, and inflammation are key factors in atherosclerosis development. Recent studies have shown senescent cells in atherosclerotic plaque secrete high levels of pro-atherogenic factors such as (matrix metalloprotein 12, matrix metalloprotein 13, interleukin 6 {IL-6}), proinflammatory cytokines, or chemokines with increased expression of senescence markers like (p53, p16, p21) [5-8].

Lifestyle changes primarily manage atherosclerosis through weight loss, blood glucose control, smoking cessation, and diet [9]. Lifestyle changes are combined with lipid-lowering pharmacotherapy and antihypertensive medications to reduce atherosclerosis's ongoing risk [9]. The most used lipid-lowering pharmacotherapy is 3-hydroxy-3-methylglutaryl coenzyme A reductase inhibitor (statin), which can be combined with adjunctive therapy, such as niacin, fibrates, and omega 3 fatty acids [9]. Managing blood pressure involves the stepwise adjustment of multiple antihypertensive medications such as beta-blockers (propranolol), calcium channel blockers (nifedipine), angiotensin-converting enzyme inhibitors (enalapril) [9]. Regardless of the current atherosclerosis management systems, mortality and morbidity appear to be accelerating, suggesting a greater need for complementary or even alternative targeted therapies in the management of atherosclerosis [1-3]. 

Despite the multiple factors involved in atherosclerosis management, there is still no cure or reliable preventive measures against atherosclerosis. A small proportion of the current range of available medications has produced adverse effects or caused harm to some patients. Sirtuins are a set of recently discovered proteins that are endogenous to the body. Sirtuins are a family of Nicotinamide Adenine Dinucleotide (NAD+)-dependent histone deacetylases (HDAC) and, thus, are referred to as class III HDAC [10-13]. They catalyze the deacetylation of lysine residues in histone and non-histone proteins [11]. An increasing number of studies suggest a possible health benefit in cardiovascular diseases via regulating cellular metabolism, cell cycle, cellular senescence, apoptosis, and genomic stability [14-17]. Aging research has made some significant inroads due to findings that overexpression of Sirtuins increases lifespan in yeast [11]. Sirtuins have been implicated in the protective effect of calorie restriction for extending life span in yeast [11]. Caloric restriction has demonstrated a marked ability to improve endothelial cell function and lower blood pressure [18].

There are seven homologs of sirtuins (SIRT 1-7) that have been cloned in humans. They require NAD to deacetylate histones [17]. Therefore, they are referred to as class III (HDAC) [17]. The seven sirtuins have diverse cellular locations [19]. SIRT1 is located in the cytoplasm and nucleus but, predominantly in the nucleus, SIRT6, and SIRT7 are located in the nucleus [19]. These three nuclear sirtuins are involved in transcription regulation, DNA repair, and inflammation [19]. SIRT2 is located in the cytosol [19]. SIRT3-5 are mainly in the mitochondria [19]. These sirtuins interact with cytosolic and mitochondrial proteins [19,20]. It has become evident that nuclear sirtuins such as SIRT1 target transcriptional regulators such as p53, FOX-O (Forks headbox O), nuclear factor-kappa B (NF-κB), PPARγ (Peroxisome proliferator-activated receptor gamma) coactivator 1α (PGC1-α) [21]. Recent reports have shown that sirtuins prevent inflammation, apoptosis, and cellular senescence in endothelial cells [19]. Also, resveratrol, which is a potent SIRT1 activator, has been reported to be vasculoprotective [21]. 

Considering the increase in morbidity and mortality related to atherosclerosis, especially among the elderly. There is a very real need to develop new therapies focused on stabilizing or even reversing atherosclerosis progression before it becomes life-threatening. Sirtuins could prove to be a good target since they are anti-inflammatory, prevent cellular senescence in endothelial cells, and are vasculoprotective. This study aims to discuss and review the mechanism in which sirtuins prevent atherosclerosis and discuss sirtuins as novel and potential drug targets against atherosclerosis. The articles used in this study were compiled from the PubMed database using the MeSH keywords "sirtuins" and "atherosclerosis,” a total of 49 articles were found. All studies included were published solely in English. We selected 45 relevant articles after a thorough assessment.

## Review

Many research papers exist investigating the beneficial effect of sirtuins in atherosclerosis via regulation of inflammation and cellular senescence in endothelial function on multiple levels. Accumulation of senescent cells within the atherosclerotic plaque increases the expression of proinflammatory cytokines. In this review, we will discuss the various types of sirtuin and their role in preventing atherosclerosis.

SIRT1 and atherosclerosis

SIRT1 plays a critical role in endothelial function by regulating various upstream factors on the genetic and biochemical level to reduce inflammation and cellular senescence [17,22-29]. SIRT1 inhibits cellular senescence by deacetylation of p53, thereby repressing it during oxidative stress or DNA damage leading to an impairment in apoptosis and cell death [17]. This property of SIRT1 indicates that it may be oncogenic. On the contrary, studies by Herranz and Serrano on mice revealed that SIRT1 is a tumor suppressor protein [22]. When a DNA is damaged, SIRT1 relocates from its bound site to the defective site to promote repair and repress transcription of the damaged genes, maintaining genomic integrity when p53 is suppressed [22]. Motta et al. carried out a study to show that SIRT1 inhibits Forkhead transcription factor FOXO3a via deacetylation and subsequently inhibits the Forkhead transcription factor-dependent apoptosis and cellular oxidative stress response [23]. They transfected human embryonic kidney cells (HEK293T) with HA-FOXO3a and transfected another sample with HA-Foxo3a and SIRT1 [23]. These precipitates were marked with anti-acetyl-lysine [23]. The samples treated with SIRT1 showed barely any trace of acetylated Foxo3a compared to the other sample suggesting SIRT1 inhibits acetylation of FOXO3a [22]. They also demonstrated a high level of FOXO3a acetylation in UV and hydrogen peroxide treated cells, thereby activating proteins involved in oxidative stress and cell death [23]. SIRT1 counteracts this effect by deacetylating FOXO3a and deactivating it [24]. In a study by Miranda et al., apolipoprotein E (Apoe−/−) mice were treated with SRT3025 (a SIRT1 activator), these mice had lower levels of plasma LDL, cholesterol, and atherosclerotic plaque compared to mice treated with placebo [24]. The study showed that SIRT1 decreases plasma LDL by reducing low-density lipoprotein receptors (Ldlr) expression and activity in hepatocytes [24]. SIRT1 achieves this by attenuating the secretion of proprotein convertase subtilisin/kexin 9 (Pcsk9), which catabolizes Ldlr in the hepatocytes before they reach the cell surface [24]. Winnik et al. demonstrated the effect of SIRT1 on atherosclerotic foam cells [25]. Wherein SIRT1 slows down foam cell formation by regulating liver-X receptors’ activity, promoting reverse cholesterol transport, stimulated by ABCA (ATP Binding Cassette Transporter) in the atherosclerotic plaque macrophages [25]. SIRT1 also has antithrombotic activity, preventing the formation of thrombosis in the carotid artery of mice by inhibition of endothelial tissue factor [25]. 

Recent studies have shown that endothelial nitric oxide synthase (eNOs) is low in senescent endothelial cells, and nitric oxide (NO) has vasodilatory, antioxidant, and atheroprotective functions [26-29]. Research by Mattagajasingh et al. showed that SIRT1 increases endothelial Nitric oxide synthase levels during caloric restriction by deacetylation of eNOs, leading to its activation and a decrease in SIRT1 levels leads to increase acetylation of eNOs in the 496 and 506 lysine residue, subsequently reducing eNOs activity [26]. Lui et al. noted that lower SIRT1 activity levels might alter autophagy regulation, and increase NF-kB levels, thus driving inflammation [27]. Autophagy is necessary to remove necrotic and old macrophages, helping slow down atherosclerosis progression [27]. Lui et al. also demonstrated that resveratrol, an activator of SIRT1, protects endothelial cells by activating adenosine 5′-monophosphate-activated protein kinase (AMPK)-SIRT1-autophagy [27]. SIRT1 inhibition leads to dysregulation of autophagy in macrophages, activating NF-kB, which drives inflammation with a build-up of autophagy markers [27]. A study by Lee et al. noted that SIRT1 regulated autophagy via deacetylation of the lysine residue on the Atg5 gene [28]. Therefore, it aids in removing aged or defective mitochondria and preventing cell death [28]. Yang et al. found that SIRT1 decreased the expression of CD40 ligand (a pro-inflammatory cytokine) in the presence of high levels of SIRT1 [29]. SIRT1 achieves this by inhibiting tumor necrosis factor-alpha (TNF-ɑ) induced CD40 activity [29].

SIRT2 and atherosclerosis

SIRT2 has been reported to stabilize atherosclerotic plaques, regulates lipid metabolism, and gluconeogenesis [30-32]. Zhang et al. demonstrated that SIRT 2 stabilizes atherosclerotic plaques by preventing macrophage polarization to the M1 phenotype, which is crucial in developing atherosclerotic plaques [30]. Furthermore, Wang and Tong reported that SIRT2 enhances lipolysis during times of fasting or caloric restriction [31]. SIRT2 achieves this by deacetylation and activation of FOXO1, stimulating the binding of FOXO1 to PPARƔ (peroxisome proliferator-activated receptor-gamma), thus suppressing the activity of PPARƔ [31]. This role of SIRT2 could prevent obesity and metabolic diseases such as diabetes, which are risk factors in atherosclerosis development [31].

Findings by Jiang et al. suggest that SIRT2 regulates gluconeogenesis during periods of fasting by deacetylation of phosphoenolpyruvate carboxykinase (PEPCK1) to stabilize it [32]. Deacetylation of PEPCK prevents its ubiquitination and degradation, then activates it to commit to the gluconeogenesis process [32].

SIRT3 and atherosclerosis

SIRT3 is primarily located in the mitochondria. It acts as an antioxidant preventing oxidative stress, which is one of the factors promoting atherosclerosis [33]. Studies by Jing et al. on mice showed that SIRT3 knockdown myoblasts have higher reactive oxygen species levels, eventually leading to a higher number of cell deaths [33]. They also observed an increased activity of superoxide dismutase and catalase in SIRT3 knockdown cells [33]. Regardless, there were still high ROS (reactive oxygen species) levels in SIRT3 knockdown cells, suggesting that SIRT3 plays a crucial role in the regulation of oxidative stress [33]. The exact mechanism is still unknown [33]. Someya et al. also revealed that SIRT3 combats oxidative stress by its deacetylation of isocitrate dehydrogenase in the tricarboxylic cycle [34]. SIRT3 activates it, leading to an increase in NADPH and a subsequent increase in reduced glutathione [34].

According to Hirschey et al., SIRT3 regulates fatty oxidation in the mitochondria during periods of caloric restriction [35]. Shimazu et al. reported on the role of SIRT3 in ketogenesis [36]. Considering the available data on SIRT3, it would not be difficult to see why a decrease in SIRT3 could lead to metabolic syndrome, increasing the risk of developing atherosclerosis.

SIRT6 and atherosclerosis

SIRT6 is involved in genomic stability, LDL cholesterol metabolism, and inflammatory stress response [37-39]. A study by Michishita et al. suggested that SIRT6 stabilizes the genome to prevent premature cellular senescence by deacetylation of histone 3 lysine9 (H3K9) residue and preventing an end to end chromatin fusion [37]. They observed that a deficiency of SIRT6 leads to premature cellular senescence due to random loss of telomere, and not necessarily the shortening of telomeres [37]. LDL cholesterol, a significant risk factor in the development of atherosclerosis, is regulated by SIRT6; as stated by Tao et al. SIRT6 inhibits Pcsk9, increasing the expression of LDLr on the cell surface and decreasing plasma LDL [38]. Repression of the NF-kB activity by SIRT6 is responsible for the anti-inflammatory and anti-aging effect of SIRT6 [38].

Kawahara et al. showed that SIRT6 alters NF-kB expression by deacetylation of H3K9 histone, halting the association between the NF-kB RELA (Rel associated protein) subunit and the promoters in its target gene, hence decreasing inflammation [39]. A combination of inflammation, cellular senescence, and high LDL cholesterol can begin a vicious cycle in atherosclerosis development, which can be stalled by SIRT6.

Other sirtuins

SIRT4 is located in the mitochondria [40]. Extensive analysis has been conducted on the function of SIRT4. Tao et al. performed an investigation to determine the role of SIRT4 in endothelial cell inflammation [40]. The results suggest overexpression of SIRT4 invitro prevents the translocation of NF-κB to the nucleus to activate the transcription of inflammatory proteins [40]. This subsequently inhibits the expression of inflammatory cytokines such as IL-6, IL-8, and the COX-prostaglandin system [40]. SIRT7 is located primarily in the nucleus [41]. Ford et al. found SIRT7 to be essential for cellular survival, activating RNA polymerase I transcription; additionally, it is seen in a high amount in metabolically active tissues [41]. SIRT7 has been shown to play a role in cardiac hypertrophy and reduced lifespan. According to Vakruhsheva et al., SIRT7 deacetylates p53, deactivating it, thus preventing apoptosis [42]. Cardiomyocytes that were deficient in SIRT7 showed extensive fibrosis and hypertrophy; this report suggests a role of SIRT7 in cell death [42]. According to a study by Zheng et al., SIRT7 inhibits vascular smooth cell proliferation and migration induced by oxidized low-density lipoprotein through the Wnt/β-catenin signaling pathway, suggesting SIRT7 inhibits atherosclerosis progression [43]. 

Not much has been studied on the role of SIRT5 on atherosclerosis. However, studies by Nagakawa et al. indicate that SIRT5 deacetylates carbamoyl phosphate synthetase 1 (CPS1), an enzyme in the urea cycle [44]. Activating CPS during fasting periods as an adaptive mechanism in eliminating ammonia waste from amino acid catabolism during periods of fasting [44]. On the other hand, Du et al. showed that SIRT5 has weak deacetylase activity with higher activity of desuccinylation and demalonylation on CPS1 [45].

Limitations and considerations

**Figure 1:**
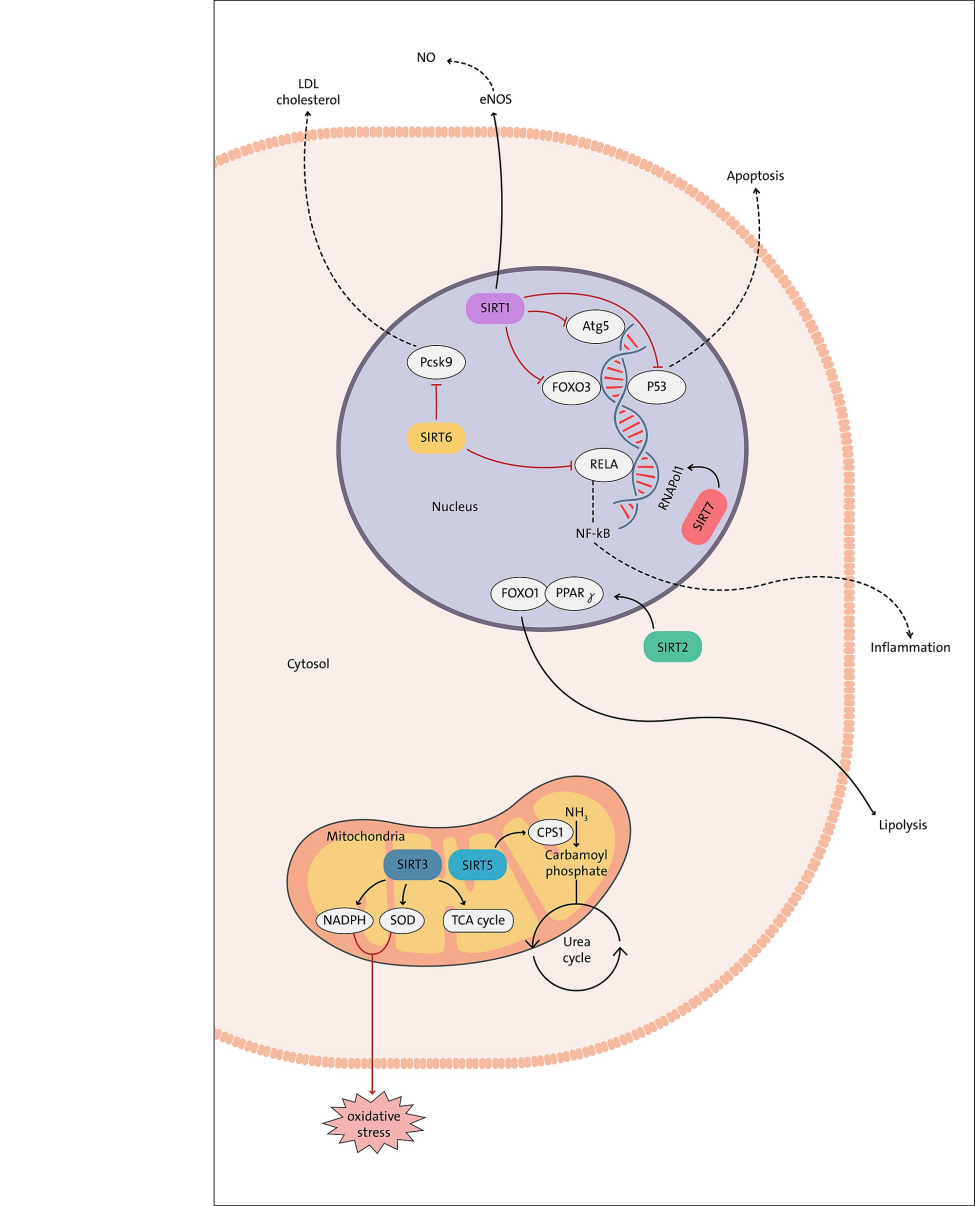
Summary of the role of sirtuins in atherosclerosis NADPH: nicotinamide adenine dinucleotide phosphate, SOD: superoxide dismutase, TCA cycle: tricarboxylic cycle, CPS1: carbamoyl phosphate synthetase I, NH3: ammonia

This is a narrative review; therefore there is no quality assessment. Most of the articles used for this review were from animal and in-vitro studies and were only written in English. The review aims to bridge the gap using available data to answer more clearly if sirtuins could help develop successful therapies for atherosclerosis. Figure 1 summarises the effect of sirtuins on atherosclerosis. Table 1 shows the relevant studies included in this review article and their findings.

**Table 1:** Relevant studies added in this review article and their findings CPS1: carbamoyl phosphate synthetase 1, HMG-CoA: hydroxy-3-methylglutaryl CoA; LDL: low-density lipoprotein; Apoe-/-: apolipoprotein E; eNOs: endothelial nitric oxide synthase; AMPK: adenosine 5′-monophosphate-activated protein kinase; TNF-α: tumor necrosis factor-alpha; PPARγ: peroxisome proliferator-activated receptor gamma; SIRT: sirtuin

Author	Year of publication	Topic	Type of study	Outcome
Herranz and Serrano [22]	2010	SIRT1: recent lessons from mouse models.	Review	Evidence shows the protective effect of SIRT1 in cardiovascular diseases
Motta et al. [23]	2004	Mammalian SIRT1 represses forkhead transcription factors	Animal study	SIRT1 regulates Fox-O. Therefore, SIRT1 has a role to play in regulating metabolism changes.
Miranda et al. [24]	2015	The SIRT1 activator SRT3025 provides atheroprotection in Apoe-/- mice by reducing hepatic Pcsk9 secretion and enhancing Ldlr expression.	Animal study	SIRT1 reduces Pcsk9 activity, which provides evidence that SIRT1 could reduce plasma LDL
Winnik et al. [25]	2012	SIRT1 an anti-inflammatory pathway at the crossroads between metabolic disease and atherosclerosis	Animal study	SIRT1 has a protective effect against thrombosis and atherogenesis in mice.
Mattagajasingh et al. [26]	2007	SIRT1 promotes endothelium-dependent vascular relaxation by activating endothelial nitric oxide synthase	Animal study	SIRT1 regulates vascular tone by deacetylation of eNOs.
Lui et al. [27]	2014	Enhancement in efferocytosis of oxidized low-density lipoprotein-induced apoptotic RAW264.7 cells through SIRT1-mediated autophagy.” International Journal of Molecular Medicine	Animal study	SIRT1 regulates autophagy of aged macrophages through the AMPK-SIRT1 pathway.
Lee et al. [28]	2008	A role for the NAD-dependent deacetylase SIRT1 in the regulation of autophagy	Animal study	They showed that SIRT1 regulates autophagy by deacetylating the Atg5 gene leading to an increase in autophagy.
Yang et al. [29]	2012	SIRT1 regulates CD40 expression induced by TNF-α via the NF-ĸB pathway in endothelial cells	In vitro study	SIRT1 anti-inflammatory effect due to its deacetylation of RelA/p65 subunit of NF-kB, which inhibits CD40 expression by TNF-ɑ induction.
Zhang et al. [30]	2018	SIRT2 decreases atherosclerotic plaque formation in low-density lipoprotein receptor-deficient mice by modulating macrophage polarization	Animal study	They showed macrophage infiltration, and apoptosis was reduced in endothelial cells with high levels of SIRT2
Wang and Tong [31]	2009	SIRT2 suppresses adipocyte differentiation by deacetylating FOXO1 and enhancing FOXO1 repressive interaction with PPARgamma	In vitro study	SIRT2 deacetylates Foxo1, stimulating its binding to PPARƔ, thus drives lipolysis
Jiang et al. [32]	2011	Acetylation regulates gluconeogenesis by promoting PEPCK1 degradation via recruiting the UBR5 ubiquitin ligase.	Animal study	SIRT2 regulates gluconeogenesis by deacetylating PEPCK
Jing et al. [33]	2011	SIRTuin-3 regulates skeletal muscle metabolism and insulin signaling via altered mitochondrial oxidation and reactive oxygen species.	Animal study	SIRT3 in skeletal muscles regulates oxidative stress, insulin resistance, and energy homeostasis, especially in type 2 diabetes mellitus.
Someya et al. [34]	2010	SIRT3 mediates the reduction of oxidative damage and prevention of age-related hearing loss under caloric restriction.	Animal study	SIRT3 increases reduced glutathione, therefore, reduces oxidative stress.
Hirschey et al. [35]	2010	SIRT3 regulates mitochondrial fatty acid oxidation by reversible enzyme deacetylation	Animal study	SIRT3 upregulates fatty acid oxidation during periods of fasting.
Shimazu et al. [36]	2010	SIRT3 deacetylates mitochondrial 3-hydroxy-3-methylglutaryl CoA synthase 2 and regulates ketone body production	Animal study	SIRT3 regulate ketone body production by deacetylation of HMG-CoA synthase.
Michishita et al. [37]	2008	SIRT6 is a histone H3 lysine 9 deacetylase that modulates telomeric chromatin.	Animal study	SIRT6 is critical in maintaining telomere integrity and chromosomal stability.
Tao et al. [38]	2013	FoxO3 transcription factor and SIRT6 deacetylase regulate low-density lipoprotein cholesterol homeostasis via control of the proprotein convertase subtilisin/kexin type 9 gene expression.	Animal study	SIRT6 regulates LDL cholesterol by modulating the expression of Pcsk9
Kawahara et al. [39]	2009	SIRT6 links histone H3 lysine 9 deacetylation to NF-kappaB-dependent gene expression and organismal life span	Animal study	SIRT6 inhibits NF-kB by interacting with histone3 lysine9 in the NF-kB site, thereby inhibiting inflammation and cellular senescence.
Tao et al. [40]	2015	SIRT4 suppresses inflammatory responses in human umbilical vein endothelial cells	In vitro study	SIRT4 combats inflammation by preventing the translocation of NF-kB into the nucleus.
Ford et al. [41]	2006	Mammalian Sir2 homolog SIRT7 is an activator of RNA polymerase I transcription.	In vitro study	SIRT7 is essential for cellular survival and RNA Pol1 transcription.
Vakhrusheva et al. [42]	2008	SIRT7 increases the stress resistance of cardiomyocytes and prevents apoptosis and inflammatory cardiomyopathy in mice.	Animal study	SIRT7 deacetylates P53 and inhibits apoptosis in cardiac myocytes.
Zheng et al. [43]	2018	SIRT7 regulates the vascular smooth muscle cells proliferation and migration via Wnt/β-catenin signaling pathway	In vitro study	SIRT7 inhibits the progression of atherosclerosis by decreasing the proliferation and migration of vascular smooth muscle cells.
Nakagawa et al. [44]	2009	SIRT5 deacetylates carbamoyl phosphate synthetase1 and regulates the urea cycle	Animal study	SIRT5 activates CPS1 during periods of fasting.
Du et al. [45]	2011	SIRT5 is a NAD dependent protein lysine demalonylase and desuccinylase.	Animal study	SIRT5 activates CPS1 mainly by demalonylation than deacetylation.

## Conclusions

Atherosclerosis is one of the primary causes of cerebral coronary and peripheral vascular diseases. This study reviews the processes involved in the development of atherosclerosis and sirtuin’s role in preventing these processes. We found that sirtuins are antiinflammatory`, prevent cellular senescence, reduce plasma LDL, and combat ROS. Sirtuins achieve this by inhibiting proinflammatory cytokines, decreasing the activity of proapoptotic genes like p53. This regulates plasma LDL cholesterol and increases the levels of reduced glutathione to reduce oxidative stress. Their multifaceted role in combating these processes involved in atherosclerosis shows evidence that sirtuins could prove useful in raising the standards for preventing and managing atherosclerosis. By using sirtuins targeted pharmacotherapies, we could potentially halt the progression of atherosclerosis before it becomes deleterious. There have been promising results in numerous preclinical studies on sirtuins during the last decade. We recommend more research into the different types of sirtuins and their role in the interplay between the endothelium and processes involved in atherosclerosis development. Further research is needed on the role of different sirtuins in atherosclerosis and their associated risk factors in humans. All current studies are based on animal trials, which is insufficient to draw a firm conclusion.
